# Joint distribution for number of crossings and longest run in independent Bernoulli observations. The R package crossrun

**DOI:** 10.1371/journal.pone.0223233

**Published:** 2019-10-01

**Authors:** Tore Wentzel-Larsen, Jacob Anhøj

**Affiliations:** 1 Centre for Child and Adolescent Mental Health, Eastern and Southern Norway, Oslo, Norway; 2 Norwegian Centre of Violence and Traumatic Stress Studies, Oslo, Norway; 3 Rigshospitalet, University of Copenhagen, Copenhagen, Denmark; University of Catania, ITALY

## Abstract

The R package crossrun computes the joint distribution of the number of crossings and the longest run in a sequence of independent Bernoulli observations. The main intended application is statistical process control where the joint distribution may be used for systematic investigation, and possibly refinement, of existing rules for distinguishing between signal and noise. While the crossrun vignette is written to assist in practical use, this article gives a hands-on explanation of why the procedures works. The article also includes a discussion of limitations of the present version of crossrun together with an outline of ongoing work to meet these limitations. There is more to come, and it is necessary to grasp the basic ideas behind the procedure implemented both to understand these planned extensions, and how presently implemented rules in statistical process control, based on the number of crossings and the longest run, may be refined.

## Introduction

The setting is defined by a number of independent observations from a Bernoulli distribution with the same success probability. In statistical process control, our main intended application, this may be the useful observations in a runchart, recording values above and below the median from previous data, disregarding any observations equal to the median [1]. The focus of the R package crossrun [2,3] is the joint distribution of number of crossings, C, and the length of the longest run, L, in random data sequences. A run is a sequence of successes or failures, delimited by a different observation or the start or end of the entire sequence. A crossing is two adjacent different observations.

**Fig 1:**
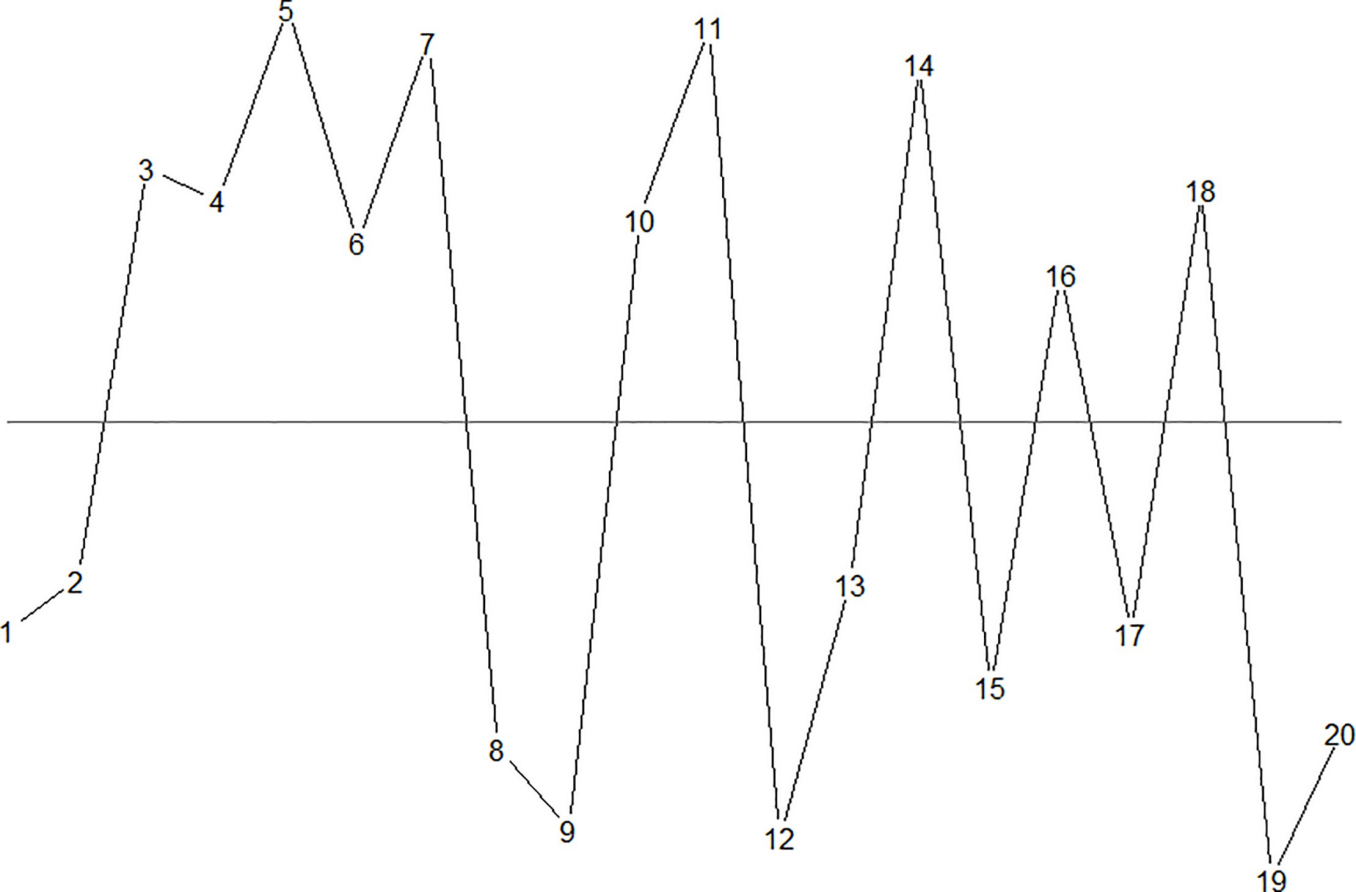
A run chart with n = 20 data points.

Fig 1 illustrates runs and crossings in a run chart with 20 random observations. Observations above and below the median represent successes and failures respectively.

The longest run consists of observations 3–7 above the median. The length of the longest run is L = 5. The number of crossings of the median is C = 10.

While the number of crossings follows a binomial distribution in the symmetric case (success probability 0.5), no closed form distribution is known for the longest run. The distribution of the longest run has been investigated in a number of articles, including Schilling [4], and Fazekas et al [5] that in fact gives recursion formulas, and approximations have been given. However, what is needed in applications is the joint distribution of these two variables, for which we are not aware of exact results. Our primary aim is to present an iterative procedure for computing this distribution, in principle for an arbitrary number of observations.

### The iterative procedure, setting

In n independent Bernoulli observations with success probability p and failure probability q = 1—p, values are denoted by 1 (success with probability p) or 0. A crossing consists of two consecutive different values, and a run of length l consists of l successive observations, delimited by a crossing or the first or last observation. The possible values of the number C of crossings are c = 0, …, n-1, and the possible values for the length L of the longest run are l = 1, …, n. The joint probabilities of L and C for given n are denoted by *P*_*n*_(*L* = *l*,*C* = *c*).

The iterative procedure involves conditioning on the first observation denoted by S, with values 1 for success (probability p) and 0 for failure (probability q). The iterative procedure computes the conditional probabilities

$$P_{n}(L=l,C=c|S=1),P_{n}(L=l,C=c|S=0)$$

This conditioning on the first observation is an essential part of the procedure. One way to see that this is reasonable is to consider the case when p is close to 1. Then most observations are successes, most runs are success runs and the conditional joint distribution of runs and crossings is quite different dependent on the first observation. It is sufficient to be able to compute these conditional distributions, because the unconditional joint distribution is

$$P_{n}(L=l,C=c)=P_{n}(L=l,C=c|S=1)\cdot p+P_{n}(L=l,C=c|S=0)\cdot q$$

For the iterative procedure to work it is also necessary to take another variable into account, the first crossing. More precisely, we denote the end position of the first crossing by F, with values F = 2, …, n. An additional value F = 1 denotes, by convention, the case of no crossing. The joint probabilities for C and L conditional on S are partitioned by further conditioning on F as detailed below.

It is important to underline that the conditioning variables S and F are not parameters of the distribution but useful constructions that help break down the iterative procedure in manageable parts.

This article first describes the setting for the iterative computation procedure and its initial stage. Next, we introduce conditioning on the starting position and partitioning on the position of the first crossing. Finally, the joint distribution of the number of crossings and the longest run conditional on these variables is given. The computation procedure is different in two cases that are subsequently described.

After the exposition of the iterative procedure follows a brief comment on the simpler symmetric case, and the precision of the procedure is discussed. The resulting joint distribution is described in a simple case, and procedures for checking are briefly commented. Next follows sections on generalizations and applications and on limitations of the procedures in crossrun together with a brief description of ongoing work to address these limitations. Finally, a Conclusions section summarizes the article. Two appendices are included, one giving details on the "times" representation in which the joint distributions are actually stored, as described in the section on precision, and one on the code for the main function crossrunbin and for the analyses in this article.

### The conditional probabilities with one observation

First, we present the starting point of the iterative procedure, the conditional probabilities in the rather redundant case with only one observation. If n = 1, then 0 is the only possible value of C and 1 the only possible value of L, therefore *P*_1_(*C* = 0,*L* = 1|*S* = 1) = *P*_1_(*C* = 0,*L* = 1|*S* = 0) = 1.

In this case the joint distribution of (C,L) is degenerate and takes the value (0,1) with probability one. Moving to more than one observation, the next step is presenting the conditional distribution of the end position F of the first crossing, conditional on the starting position S.

### The distribution of the first crossing conditional on the starting position

If the first value is 1 (success), no crossing means that all the remaining n-1 values are also 1, therefore *P*_*n*_(*F* = 1|*S* = 1) = *p*^*n*−1^. Similarly, *P*_*n*_(*F* = 1|*S* = 0) = *q*^*n*−1^. Next, if f = 2, …, n and the first value is a success, F = f means that the sequence starts with a success, then f-2 more successes and then one failure. Therefore,

$$P_{n}(F=f|S=1)=p^{f-2}\cdot q,P_{n}(F=f|S=0)=q^{f-2}\cdot p,f=2,\ldots ,n$$

where the last formula is based on a similar argument conditional on S = 0. In the following, arguments will in many cases be given for S = 1 only, and similar results for S = 0 will be stated with no explicit arguments. By symmetry, these results will simply involve replacing p by q. In the next step the formulas in this section will be used for partitioning the joint conditional probabilities for C and L given S, by the position F of the first crossing.

### Partitioning by the position of the first crossing

Partitioning on F we have

$$P_{n}(L=l,C=c|S=1)=\sum_{f=1}^{n}P_{n}(L=l,C=c|S=1,F=f)\cdot P_{n}(F=f|S=1)$$

where, as shown in the previous section,

$$P_{n}(F=f|S=1)=p^{f-2}q\;\mathrm{if}\;f\geq 2\;\mathrm{and}\;P_{n}(F=1|S=1)=p^{n-1}$$

The formulas for *P*_*n*_(*L* =*l*,*C* = *c*|*S* = 0) are the same, just interchanging p and q. This implies that the joint probabilities of C and L conditional on S may be computed if it is possible to compute all the joint probabilities of C and L conditional on S and F. This is the next step.

### Joint distribution conditional on starting position and first crossing

First, if there is no crossing (F = 1) the entire sequence constitutes one single run, therefore

$$P_{n}(C=0,L=1|S=1,F=1)=P_{n}(C=0,L=1|S=0,F=1)=1$$

and all other conditional probabilities are 0. Thus, the matrices of joint probabilities of C and L conditional on F = 1 together with each value of S, are matrices with all components equal to 0, except for a 1 in the upper right corner.

If crossings do occur (f = 2, …, n), the conditional probabilities

$$P_{n}(C=c,L=l|S=1,F=f),P_{n}(C=c,L=l|S=0,F=f)$$

are more complicated. The key to computing these probabilities is to recognize that, except for the initial run of f-1 observations, the remaining observations constitute n-(f-1) = n+1-f identical and independent Bernoulli observations with success probability p, they represent the same setting as for all n observations, just a shorter sequence. Further, these n+1-f observations are also conditional on a fixed value of their first observation, only that this fixed value is the opposite as in the entire sequence. This is because the last n+1-f observations start with the observation after the first crossing.

We now have to distinguish between two cases. In case 1, the f-1 observations in the initial run, before the first crossing, are at least as many as the last n+1-f ones. In case 2, the initial run is shorter:

Case 1: f-1 ≥ n+1-f

Case 2: f-1 < n+1-f

**Fig 2:**
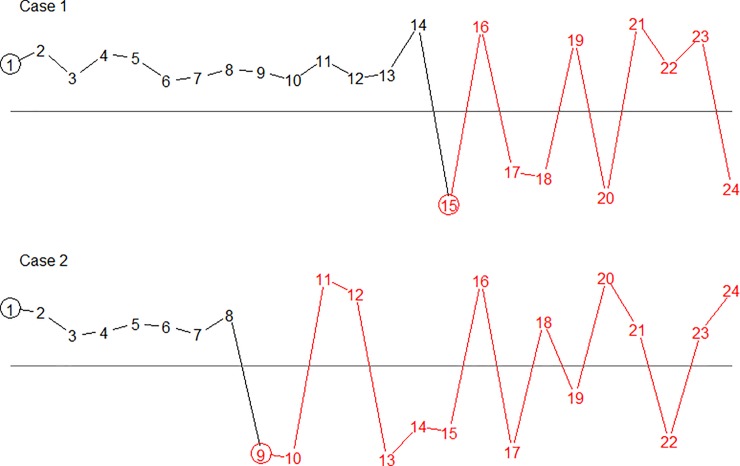
Two runcharts with n = 24 data points, both starting above the median.

The two cases are illustrated in Fig 2 below. In both cases the observations (red) from the end of the first crossing constitute a runchart of its own, only shorter and starting on the opposite side. In Case 1 (top), the initial run of f-1 = 14 observations is longest, l = f-1 = 14. The number of crossings in the last n+1-f = 10 observations is between 0 and 9, and the total number of crossings is one more, 1 ≤ c ≤ 10. In Case2 (bottom) the initial run of f-1 = 8 observations may or may not be longest, l ≥ 8. It is a longest run precisely if no run within the last n+1-f = 16 (red) observations is longer than 8. If all observations in the last 16 observations are below the median, l = 16, thus for the whole sequence 8 ≤ l ≤ 16. The number of crossings in the last part (red, 16 observations) is between 0 and 16–1 = 15, and the number of crossings in the whole sequence is one more, 1° ≤ c ≤ 16. What is shown here in an example is the core of the general argument that follows.

Top: f = 15, the initial run is necessarily longest. Bottom: f = 9, the initial run may or may not be longest.

### Case 1, at least as many observations before the first crossing as thereafter

This is the simplest case. Here, the first f-1 observations constitute a run of length f-1, and no run in the last n+1-f observations may be longer than that. Therefore, the longest run is f-1, and the non-zero probabilities *P*_*n*_(*C* = *c*,*L* = *l*|*S* = 1,*F* = *f*) are confined to the vertical strip l = f-1. And, in fact, to only a part of this strip. First, there is at least one crossing, from time f-1 to f. Also, any further crossings are within the last observations, and may be any number between 0 and (n+1-f)– 1. The total number of crossings may therefore be any number between 1 and n+1-f, which means that the non-zero probabilities *P*_*n*_(*C* = *c*,*L* = *l*|*S* = 1,*F* = *f*) are confined to the strip l = f-1, 1 ≤ c ≤ n+1-f.

The non-zero probabilities *P*_*n*_(*C* = *c*,*L* = *l*|*S* = 1,*F* = *f*), *l* = *f*−1,1≤*c*≤*n*+1−*f* are somehow determined by what happens within the last n+1-f observations. More specifically, the last n+1-f observations constitute a sequence of the same type as the original n observations, only shorter, and with the starting observation fixed on the opposite side of the central line. To put it into a formula,

$$P_{n}(C=c,L=f-1|S=1,F=f)=P_{n+1-f}(C=c-1|S=0)$$

where C = c-1 is because the crossing from f-1 to f is just before the last n+1-f observations. Similarly,

$$P_{n}(C=c,L=f-1|S=0,F=f)=P_{n+1-f}(C=c-1|S=1)$$

The probabilities on the right-hand side of these formulas are for a lower number of observations and are therefore already computed in the iterative procedure.

Note that the n+1-f observations after the initial run start on opposite side of the centre line. Therefore, it is necessary to compute conditional probabilities conditional on starting values both above and below the centre line in the iterative procedure, they cannot be computed separately. The computations are a bit more complicated in the second case, when the initial run is the shorter part, but the main idea is the same.

### Case 2, fewer observations before the first crossing than thereafter

As in case 1, the total number of crossings is between 1 and n+1-f. As to the longest run L, it cannot be shorter than f-1 or longer than n+1-f, and it is necessary to distinguish between values l = f-1 and l ≥.f. A longest run f-1 in the entire sequence means that all runs in the last n+1-f observations have length l ≤ f-1. Therefore

$$P_{n}(C=c,L=f-1|S=1,F=f)=P_{n+1-f}(C=c-1,L\leq f-1|S=0)$$

(and similarly conditional on S = 0). For longer runs in which f ≤ l ≤ n+1-f, the longest run has to be within the last n+1-f observations and we have

$$P_{n}(C=c,L=l|S=1,F=f)=P_{n+1-f}(C=c-1,L=l|S=0)$$

(and similarly conditional on S = 0). All these conditional probabilities, based on a shorter sequence, have already been computed in an iterative computation procedure.

### Simplifications in the symmetric case

For p = 0.5 there is a symmetry between crossings up or down, and between success and failure runs. Therefore, conditioning on the first observation is not necessary, although it is still necessary to partition on the first crossing F. Also, by an induction argument following the iterative procedure, all these probabilities are integer multiples of 0.5^*n*−1^ and, in fact, represent a partition of the binomial coefficients in the distribution of C, by the values l = 1, …, n of L.

### Precision considerations

To enhance precision, computations have been performed in the R package Rmpfr [6], an R interface to the GNU MPFR library [7]. Preliminary investigations pointed to precision problems above values about 50 for sequence length n without this increased precision, but no such problems up to n = 100 when using Rmpfr. To further enhance precision, probabilities are represented in the times representation, they have been multiplied by m^n-1^ where m is a multiplier with default value 2. Thereby very small numbers are avoided, at least to some extent, and the numbers computed are integers in the symmetric case. The joint probabilities, using the times representation, in the symmetric case for n = 16 are shown in Table 1 below in this representation:

**Table 1:** Joint distribution in the symmetric case, n = 16. Times representation.

	l = 1	2	3	4	5	6	7	8	9	10	11	12	13	14	15	16
c = 0	0	0	0	0	0	0	0	0	0	0	0	0	0	0	0	1
1	0	0	0	0	0	0	0	1	2	2	2	2	2	2	2	0
2	0	0	0	0	0	6	15	21	18	15	12	9	6	3	0	0
3	0	0	0	1	34	90	106	84	60	40	24	12	4	0	0	0
4	0	0	0	65	300	370	280	175	100	50	20	5	0	0	0	0
5	0	0	21	525	960	741	420	210	90	30	6	0	0	0	0	0
6	0	0	266	1652	1617	882	392	147	42	7	0	0	0	0	0	0
7	0	1	1106	2716	1652	672	224	56	8	0	0	0	0	0	0	0
8	0	36	2268	2646	1080	324	72	9	0	0	0	0	0	0	0	0
9	0	210	2640	1605	450	90	10	0	0	0	0	0	0	0	0	0
10	0	462	1815	605	110	11	0	0	0	0	0	0	0	0	0	0
11	0	495	726	132	12	0	0	0	0	0	0	0	0	0	0	0
12	0	286	156	13	0	0	0	0	0	0	0	0	0	0	0	0
13	0	91	14	0	0	0	0	0	0	0	0	0	0	0	0	0
14	0	15	0	0	0	0	0	0	0	0	0	0	0	0	0	0
15	1	0	0	0	0	0	0	0	0	0	0	0	0	0	0	0

The corresponding joint probabilities are obtained by dividing these integers by 2^*n*−1^ = 2^15^ = 32768, for instance *P*_16_(*C* = 5,*L* = 6) = 741/32768 = 0.023. The highest joint probability is *P*_16_(*C* = 7,*L* = 4) = 2716/32768 = 0.083. It is also seen that a high proportion of the joint probabilities consists of zeroes, and except for some very small numbers the joint probabilities are concentrated within a narrow sloping band, as also commented in the crossrun vignette, these are fairly general phenomena. For comparison the joint distribution for n = 16 is also shown in Table 2 below for p = 0.6, a case where observations tend to stay above the midline. These probabilities are still shown in the "times" representation, they are multiplied by 2^*n*−1^ = 32768, and are shown with one decimal digit:

**Table 2:** Joint distribution for p = 0.6, n = 16. Times representation.

p = 0.6	l = 1	2	3	4	5	6	7	8	9	10	11	12	13	14	15	16
c = 0	0.0	0.0	0.0	0.0	0.0	0.0	0.0	0.0	0.0	0.0	0.0	0.0	0.0	0.0	0.0	9.3
1	0.0	0.0	0.0	0.0	0.0	0.0	0.0	0.7	1.6	1.9	2.6	3.8	5.6	8.3	12.4	0.0
2	0.0	0.0	0.0	0.0	0.0	7.5	22.8	41.2	39.3	37.5	35.3	31.9	26.2	16.5	0.0	0.0
3	0.0	0.0	0.0	0.7	28.0	88.6	130.0	121.0	102.2	82.8	61.6	38.9	16.6	0.0	0.0	0.0
4	0.0	0.0	0.0	63.4	337.8	485.0	423.3	302.3	202.2	120.6	58.5	18.0	0.0	0.0	0.0	0.0
5	0.0	0.0	15.9	451.3	947.6	845.0	550.2	323.0	166.1	67.6	16.7	0.0	0.0	0.0	0.0	0.0
6	0.0	0.0	234.2	1619.3	1784.1	1098.1	557.9	245.0	83.5	16.8	0.0	0.0	0.0	0.0	0.0	0.0
7	0.0	0.7	900.4	2439.2	1660.7	764.3	295.9	87.9	15.2	0.0	0.0	0.0	0.0	0.0	0.0	0.0
8	0.0	28.7	1977.6	2518.8	1138.4	386.4	99.8	14.8	0.0	0.0	0.0	0.0	0.0	0.0	0.0	0.0
9	0.0	160.0	2159.1	1427.7	444.0	101.6	13.2	0.0	0.0	0.0	0.0	0.0	0.0	0.0	0.0	0.0
10	0.0	369.8	1535.6	553.4	111.8	12.8	0.0	0.0	0.0	0.0	0.0	0.0	0.0	0.0	0.0	0.0
11	0.0	379.0	582.9	114.6	11.7	0.0	0.0	0.0	0.0	0.0	0.0	0.0	0.0	0.0	0.0	0.0
12	0.0	223.9	127.4	11.5	0.0	0.0	0.0	0.0	0.0	0.0	0.0	0.0	0.0	0.0	0.0	0.0
13	0.0	68.2	10.9	0.0	0.0	0.0	0.0	0.0	0.0	0.0	0.0	0.0	0.0	0.0	0.0	0.0
14	0.0	11.3	0.0	0.0	0.0	0.0	0.0	0.0	0.0	0.0	0.0	0.0	0.0	0.0	0.0	0.0
15	0.7	0.0	0.0	0.0	0.0	0.0	0.0	0.0	0.0	0.0	0.0	0.0	0.0	0.0	0.0	0.0

Probabilities are multiplied by 2^*n*−1^ = 32768 (times representation) and shown with one decimal. Even in the times representation the probabilities are not represented with integers in non-symmetric cases.

## Generalization and applications

The iterative procedure has been generalized to time series where the success probability may vary, as long as the observations are assumed to be independent. This is implemented in a function crossrunchange. Here, the success probability is replaced by a sequence of probabilities, one for each observation.

The joint distributions for the number of crossings and the longest run may be used to investigate, and possible refine, the Anhoej rules in statistical process control ([8], Table 1). The Anhoej rules employ two tests for non-random variation in data over time:

Shift rule: unusually long runs of consecutive data points on the same side of the centre line. This rule is triggered if there are one or more runs longer than *log*_2_(*n*)+3 rounded to the nearest integer, where n = the number of data points not on the median.

Crossings rule: the curve crosses the centre line unusually few times. This rule is triggered if there are fewer crossings than the lower 5th percentile from the cumulative binomial distribution function with a success probability of 0.5 and n-1 trials.

While these rules have shown to be useful, as seen when preparing our recent article [9] they do not have quite monotone sensitivities and specificities. For such work access to the exact joint distributions has decisive advantages compared to simulations. We are at present working on calculation of exact values of sensitivities and specificities of the Anhoej rules, and also on a modification of the rules with less variation in sensitivities and specificities. Preliminary investigations indicate that this modification may improve the practical usefulness of the Anhoej rules.

To illustrate the use of the joint distributions of C and L we present in Table 3 below the specificities of the Anhoej rules for sequence lengths between 10 and 100 in the following table.

**Table 3:** Specificities of the Anhoej rules.

Specificities of the Anhoej rules										
	Sequence lengths represented as tens (columns) + ones (rows)									
	10	20	30	40	50	60	70	80	90	100
0	0.955	0.929	0.936	0.921	0.927	0.926	0.908	0.907	0.891	0.929
1	0.951	0.933	0.920	0.910	0.935	0.915	0.914	0.898	0.931	
2	0.957	0.917	0.929	0.915	0.923	0.922	0.904	0.904	0.922	
3	0.963	0.952	0.935	0.903	0.931	0.911	0.911	0.894	0.929	
4	0.939	0.934	0.922	0.910	0.918	0.919	0.916	0.901	0.920	
5	0.949	0.944	0.929	0.897	0.927	0.924	0.908	0.906	0.927	
6	0.953	0.950	0.915	0.936	0.933	0.915	0.913	0.898	0.933	
7	0.935	0.936	0.922	0.943	0.923	0.921	0.904	0.903	0.925	
8	0.941	0.943	0.908	0.932	0.929	0.911	0.910	0.894	0.931	
9	0.921	0.928	0.916	0.939	0.919	0.918	0.901	0.900	0.922	

The sequence lengths are represented as tens in columns and ones in rows. For instance, the specificity is 0.897 for sequence length 45 = 40+5 and 0.936 for sequence length 46. The specificities may also be estimated by extensive and complicated simulations as used in [9], but with lower precision.

## Limitations and planned extensions

There are at present two main limitations. First, in applications in statistical process control when observations are categorized as above or below a midline, the iterative procedure presupposes that the midline is determined from previous data, usually the median. If the midline is the median in the same data set the procedure does not apply since subsequent observations are no longer fully independent. Work is, however, underway to tackle this case. Briefly, the median divides the useful observations (observations not on the median, see [8] for details) into two parts of equal size. The useful observations are then necessarily an even number, say n = 2m, the useful observations above the median is a subset of size m, and all such subsets are equally probable. To find the number of such subsets for each combination of the number of crossing and the longest run is in fact tractable if it is generalized to all subsets, not necessarily of size m = n/2, and an iterative procedure resembling the procedure implemented in the function crossrunbin has been developed. The procedure, due to the large number of such subsets, is more demanding in terms of computation time and storage requirements, and it has so far only been possible to use it up to n = 64. Specifically, using a PC with an Intel Core i5 processor and 8 GB RAM computation times for n = 10, 20 and 40 were 3 seconds, 27 seconds and 9 minutes and storage requirements were 9 kB, 107 kB and 2MB, respectively. Preliminary investigations seem to indicate that the difference from the case of a predetermined midline is smaller for longer sequences. A function crossrunem has been written for this case and is planned for inclusion in an update of the package crossrun.

Another important limitation is that the iterative procedure does not apply for autocorrelated time series. Here also, work is in progress in a simple autocorrelation model in which the probabilities of "success" and "failure" in each observation may depend on the previous observation only, and a function crossrunauto is planned for inclusion in a future update. The practical value of this procedure is likely to be limited since decision rules based on the number of crossings and the longest run are probably not particularly useful for autocorrelated series, but at least the procedure may be used to investigate the extent of the problem.

A third limitation is that the code has so far only been checked for small n. For applications in statistical process control this should not be a problem, therefore details above, and in the crossrun vignette, stop at n = 100. We have now extended the computations up to n = 200 both in the symmetric case and for probability 0.8, and the procedure seems to work well, except that it was necessary to increase the mpfr precision parameter from the default value of 120 to 240 bits. Then the marginal distributions of *C* in the symmetric case agreed completely with the binomial coefficients for all n up to 200. The sum of the joint distribution for probability 0.8, in the times representation, agreed with 2^*n*−1^, with discrepancies in the 11. decimal or smaller. It was also checked, both in the symmetric case and for probability 0.8, that the mean of C·L, an example of a demanding calculation, agreed well with simulation results for n = 200. Details are included at the end of the article script. Still, as expected this points to increasing precision demands for obtaining accurate joint distributions for longer sequences. The procedures implemented in crossrun are therefore not likely to be practically feasible for applications that require very long sequences.

## Conclusions

The crossrun package includes functions for computing the probabilities of the joint distribution of longest run and number of crossings in random data series. To our knowledge, this distribution has not been studied before.

The ability to calculate exact probabilities for the joint distribution allows for the development of better prediction limits for longest run and crossings in random data series. In turn, this allows for better separation of signal and noise, i.e. random and non-random variation in statistical process control and possibly also in other areas of application.

## Appendix 1: Details on the times representation

The times representation of the joint distribution is defined as

$${\mathrm{Pt}}_{n}(C=c,L=l|S=1)=m^{n-1}\cdot P_{n}(C=c,L=l|S=1)$$

where m is a multiplier normally set as m = 2. The case of conditioning on S = 0, starting below the midline, is similar. Denoting the success probability as p and the failure probability as q = 1-p the main decomposition for probabilities is

$$\begin{array}{c} P_{n}(C=c,L=l|S=1)=p^{n-1}\cdot P_{n}(C=c,L=l|S=1,F=1)+\\ \sum_{f=2}^{n}p^{f-2}\cdot q\cdot P_{n}(C=c,L=l|S=1,F=f) \end{array}$$

where the first term conditions on F = 1, by convention corresponding to no crossing, and each of the remaining terms conditions on F = f, f = 2, …, n corresponding to the first crossing from f-1 to f. The corresponding decomposition in the times representation may be written as

$$\begin{array}{c} {\mathrm{Pt}}_{n}(C=c,L=l|S=1)=\\ \sum_{f=2}^{n}\left(pm{)}^{f-2}\cdot qm\cdot m^{n-f}\cdot P_{n}(C=c,L=l|S=1,F=f\right) \end{array}$$

In the first term for no crossing the probability is simply 1 if C = 0, L = n, and 0 otherwise. This term is the same in the times representation except for the initial factor in which *p*^*n*−1^ is replaced by (*pm*)^*n*−1^. In the symmetric case both pm and qm are equal to 1, thus the initial factor is 1 and the term as a whole is actually simpler in the times representation in the symmetric case.

In the remaining terms, for the first crossing from f-1 to f, there is first a factor that is similar to the corresponding factor in the original representation, except that p is replaced by pm and q by qm. Again, this first factor is actually equal to 1 in the symmetric case. The rest of the term is *m*^*n*−*f*^·*P*_*n*_(*C* = *c*,*L* = *l*|*S* = 1,*F* = *f*). As shown previously, the probabilities *P*_*n*_(*C* = *c*,*L* = *l*|*S* = 1,*F* = *f*) are determined by the joint distribution *P*_*n*+1−*f*_(*C* = *c*′,*L* = *l*′|*S* = 0) for the observations starting at the end of the first crossing. Here we condition on the opposite starting value S = 0 since this last part of the sequence starts just where the first crossing has occurred. As to the main part of the term *P*_*n*_(*C* = *c*,*L* = *l*|*S* = 1,*F* = *f*) we more specifically have seen that it is determined by the joint distribution *P*_*n*+1−*f*_(*C* = *c*′,*L* = *l*′|*S* = 0) by simple operations in terms of additions and reshuffling only. The corresponding joint distribution in the times representation is

$${\mathrm{Pt}}_{n+1-f}(C=c\prime ,L=l\prime |S=0)=m^{(n+1-f)-1}\cdot P_{n+1-f}(C=c\prime ,L=l\prime |S=0)$$

where the leading factor *m*^(*n*+1−*f*)−1^ = *m*^*n*−*f*^ is exactly the same as the leading factor in *m*^*n*−*f*^·*P*_*n*_(*C* = *c*,*L* = *l*|*S* = 1,*F* = *f*). Therefore, the term

$$m^{n-f}\cdot P_{n}(C=c,L=l|S=1,F=f)$$

is determined by the joint distribution

$${\mathrm{Pt}}_{n+1-f}(C=c\prime ,L=l\prime |S=0)$$

on the times scale by exactly the same additions and reshufflings as apply on the original probability scale.

## Appendix 2: R code for the iteration procedure

The iterative procedure is coded in the function crossrunbin, and is available by typing

library(crossrun)

?crossrunbin

within R, after having installed the package crossrun. R code for all analyses in this article is available in the S1 File crossrunart.R. The R packages Rmpfr and crossrun should have been installed before running these analyses.

## Supporting information

S1 File R script for all analyses in the article. (R)
